# Monometallic and Bimetallic Catalysts Supported on Praseodymium-Doped Ceria for the Water–Gas Shift Reaction

**DOI:** 10.3390/molecules28248146

**Published:** 2023-12-18

**Authors:** Weerayut Srichaisiriwech, Pannipa Tepamatr

**Affiliations:** Department of Chemistry, Faculty of Science and Technology, Thammasat University, Pathumthani 12120, Thailand; weerayut@tu.ac.th

**Keywords:** Re, hydrogen production, water–gas shift, bimetallic catalyst

## Abstract

The water–gas shift (WGS) performance was investigated over 5%Ni/CeO_2_, 5%Ni/Ce_0.95_Pr_0.05_O_1.975_, and 1%Re4%Ni/Ce_0.95_Pr_0.05_O_1.975_ catalysts to decrease the CO amount and generate extra H_2_. CeO_2_ and Pr-doped CeO_2_ mixed oxides were synthesized using a combustion method. After that, Ni and Re were loaded onto the ceria support via an impregnation method. The structural and redox characteristics of monometallic Ni and bimetallic NiRe materials, which affect their water–gas shift performance, were investigated. The results show that the Pr addition into Ni/ceria increases the specific surface area, decreases the ceria crystallite size, and improves the dispersion of Ni on the CeO_2_ surface. Furthermore, Re addition results in the enhancement of the WGS performance of the Ni/Ce_0.95_Pr_0.05_O_1.975_ catalyst. Among the studied catalysts, the ReNi/Ce_0.95_Pr_0.05_O_1.975_ catalyst showed the highest catalytic activity, reaching 96% of CO conversion at 330°. It was established that the occurrence of more oxygen vacancies accelerates the redox process at the ceria surface. In addition, an increase in the Ni dispersion, Ni surface area, and surface acidity has a positive effect on hydrogen generation during the water–gas shift reaction due to favored CO adsorption.

## 1. Introduction

Water–gas shift (WGS) reaction is an industrial technology that involves the reaction of water vapor and carbon monoxide to generate H_2_ and CO_2_. The water–gas shift reaction takes place according to the following equation:

CO + H_2_O ⇆ H_2_ + CO_2_         ΔH_298_ = −41.2 kJ/mol (1)

The development of H_2_ and fuel cell technologies provides numerous advantages such as high-efficiency power, environmentally friendliness, and sustainability. WGS reactions have received widespread attention to increase the H_2_ concentration in the syngas. Hydrocarbon reforming can produce syngas that consists of CO, H_2_, H_2_O, and CO_2_. However, a trace concentration of CO poisons the catalysts utilized in fuel cells [1]. Precious metal-based proton exchange membrane fuel cell anodes require a carbon monoxide amount in the inlet gas below 10–20 ppm; otherwise, the anode is poisoned [2]. A purification process is required to decrease the CO concentration lower than the cell tolerance level after hydrogen is produced via the reforming process of carbon-containing molecules (such as hydrocarbons or alcohols). The advantage of using the WGS reaction is to reduce CO content while generating more hydrogen as fuel for the H_2_ fuel cell. The appearance of a suitable catalyst in the WGS reaction can reduce the CO content to 10 ppm.

The support plays a critical role in oxidation reactions such as water–gas shift reactions or CO oxidation. The utilization of redox-active oxides such as CeO_2_ as a support material leads to superior catalytic efficiency compared to the use of other oxide support such as alumina or silica [3,4,5,6]. Due to its redox characteristics, CeO_2_ can promote vacancy generation and water dissociation, which plays an important role in the catalytic performance of water–gas shift reactions. However, pure CeO_2_ has some disadvantages, like deactivation of the Ce^4+^/Ce^3+^ redox couple and thermal sintering, resulting in a reduction in its oxygen storage capacity (OSC) and catalytic performance [7]. Thus, many efforts have been dedicated to improving the reducibility and catalytic performance of CeO_2_ by doping it with other cations to generate more oxygen vacancies and develop its resistance to thermal sintering [8,9]. In this part, doping CeO_2_ with aliovalent (such as Eu^3+^, La^3+^, Gd^3+^, and Sm^3+^) cations [10,11,12] or isovalent (such as Zr^4+^, Hf^4+^, and Ti^4+^) cations [13,14] has been well described in the literature. The incorporation of an aliovalent cation into the CeO_2_ lattice produces oxygen vacancies by charge compensation on the final oxide materials [15]. Conversely, the CeO_2_ doping with isovalent cations also results in the enhancement of the redox properties of CeO_2_. Therefore, the partial substitution of Ce^4+^ by Zr^4+^ causes a deformation in the CeO_2_ lattice because of the lower ionic radius of isovalent cations such as Zr^4+^, deriving an enhancement in the OSC of CeO_2_. Moreover, these two effects can also be combined in a single study when doping CeO_2_ with variable oxidation states of lanthanide elements. In such cases, Tb [16] and Pr [17,18] are the most studied ones. The Pr addition into the CeO_2_ lattice enhances both oxygen desorption and oxygen vacancy generation compared to pure CeO_2_. This result is due to the lower binding energy of oxygen anions in Ce-Pr mixed oxides and the higher reduction potential of Pr^4+^/Pr^3+^ compared to Ce^4+^/Ce^3+^. Therefore, the Zr dopant induces a limited amount of oxygen vacancies into the CeO_2_ lattice [19,20], whereas the Pr dopant leads to a greater concentration of oxygen vacancies [21]. These behaviors result in redox properties in the Ce-Pr mixed oxides being superior to those of other mixed oxides. In previous works, Ce-Pr-O mixed oxide supports were studied for WGS reaction. It was found that the optimum promoting effect of Pr appears at a low loading of 5 %wt. [22,23]. In addition, the catalytic activity of such oxides can be obviously improved by adding a small amount of transition metals (cobalt, chromium, copper, and nickel) [24,25,26]. Nickel combined with CeO_2_-based oxides are cost-effective alternatives to expensive noble metal catalysts and are often more reactive than noble metals [27]. However, CO and CO_2_ methanation are very common side reactions for these catalysts [28,29]. This behavior is usually tempered by the incorporation of a second active metal like platinum [30,31]. Rhenium might be a good choice for replacing Pt catalyst because of its good electrochemical properties, its cheaper price compared to platinum, and its sustainable source. Re is widely used as a second metal to form bimetallic catalysts. In recent years, the use of bimetallic catalysts has attracted much attention due to their excellent efficiency and capability [32,33,34]. The effect of rhenium on the WGS performance of Pt/TiO_2_ and Pt/ZrO_2_ catalysts has been investigated, and the results exhibited that rhenium addition induces an increase in the WGS activity of Pt catalysts. Rhenium acts as an anchor for the platinum particles to enhance the Pt dispersion. In addition, the redox process between Re^4+^ and Re^7+^ over the WGS reaction would promote CO oxidation on the Pt catalyst [35,36]. Our initial studying found that rhenium also enhanced the WGS activity when it was doped on Ni/CeO_2_–based oxides [11,37,38]. Apart from enhancing the performance of the WGS reaction, Pt–Re/carbon has also been reported to be active in several reactions, such as the reforming process [23] and glycerol to syngas conversion [39]. Furthermore, in studying the catalytic activity of ReCo/Al_2_O_3_ for the Fischer–Tropsch reaction found that Re addition increased the reaction rate of the Co catalyst. Re has been shown to be a good promoter by facilitating the reduction rate of cobalt species and producing more available active Co metal sites to participate in the reaction [40].

In this work, the performance of Ni/CeO_2_ and Ni/Ce_0.95_Pr_0.05_O_1.975_ catalysts for the water–gas shift reaction was studied. Additionally, the role of rhenium addition on the water–gas shift performance of Ni/Ce_0.95_Pr_0.05_O_1.975_ catalyst was also observed. Therefore, the utilization of Pr as a dopant and Re as a metal additive in this work to maximize the WGS performance. The physicochemical properties of monometallic and bimetallic catalysts were examined to clarify the key factors in increasing the catalytic activity using the following techniques: X-ray diffraction, BET surface area, NH_3_ temperature programmed desorption, H_2_ temperature programmed reduction, Raman spectroscopy, and chemisorption techniques. 

## 2. Results and Discussion

### 2.1. Catalysts Characterization

**Figure 1:**
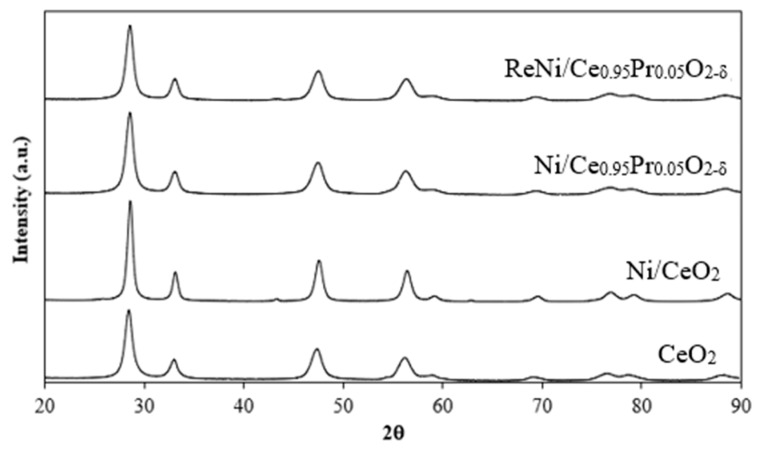
XRD patterns of Ni catalysts and CeO_2_ support.

X-ray diffraction patterns of CeO_2_, 5%Ni/CeO_2_, 5%Ni/Ce_0.95_Pr_0.05_O_1.975_, and 1%Re4%Ni/Ce_0.95_Pr_0.05_O_1.975_ were illustrated in Figure 1. It suggests that the diffraction peaks of all catalysts correspond well to CeO_2_ phases with a cubic structure (Joint Committee on Powder Diffraction File No. 43-1002). In addition, the weak peaks at 2θ about 37.1°, 43.2° and 63.2° attributed to the NiO phases of Ni-based catalysts, suggesting that there was a small proportion of nickel oxide. The CeO_2_ crystallite size of supported Ni catalysts was determined using the Debye–Scherrer equation (Table 1). The calcination of Ni/CeO_2_ catalyst at a high temperature (650 °C) after impregnation of Ni onto ceria support leads to the aggregation of ceria crystallites; thereby, the surface area decreases with a growth in CeO_2_ crystallite size. However, Pr addition into 5%Ni/CeO_2_ results in a decrease in the ceria crystallite size together with an increase in a specific surface area. The diffraction peaks of Ni/Ce_0.95_Pr_0.05_O_1.975_ and ReNi/Ce_0.95_Pr_0.05_O_1.975_ appeared at lower diffraction angles compared with the diffraction peaks of Ni/CeO_2_, indicating that Pr incorporation in the CeO_2_ lattice enlarged unit cell. The enhancement of the unit cell for Ni/Ce_0.95_Pr_0.05_O_1.975_ when compared with Ni/CeO_2_ is due to Ce^4+^ ions (0.097 nm) being replaced by larger Pr^3+^ ions (0.112 nm). Therefore, an oxygen vacancy is expected to be formed because Pr^3+^ incorporation in the CeO_2_ lattice produces unbalanced charges and strain. On the other hand, the diffraction peaks of Ni/CeO_2_ appeared at higher diffraction angles compared with the diffraction peaks of pure CeO_2_ because of the lattice contraction after calcination at high temperatures. Nickel could not be incorporated into CeO_2_ lattice due to the nature of impregnation synthesis but the reduction in the cell dimension because of the decomposition of surface hydroxyls during calcination at 650 °C.

H_2_ chemisorption analysis was used to determine the Ni dispersion of supported Ni catalysts. It was found that an addition of Pr to Ni/CeO_2_ increases Ni dispersion on the catalyst surface. Moreover, rhenium impregnation onto Ni/Ce_0.95_Pr_0.05_O_1.975_ tremendously enhanced the dispersion and surface area of metallic nickel. This result may be due to the movement of electrons between Re, Ni, and CeO_2_, which results in the formation of strong interaction between Ni metal and support; thereby, the metal dispersion and metal surface coverage enhances, whereas particle size reduces. 1%Re4%Ni/Ce_0.95_Pr_0.05_O_1.975_ exhibited the highest Ni surface area and dispersion among all the catalysts. Usually, a greater metal surface area provides more surface active sites exposed to reactants [36,37]. 

**Figure 2:**
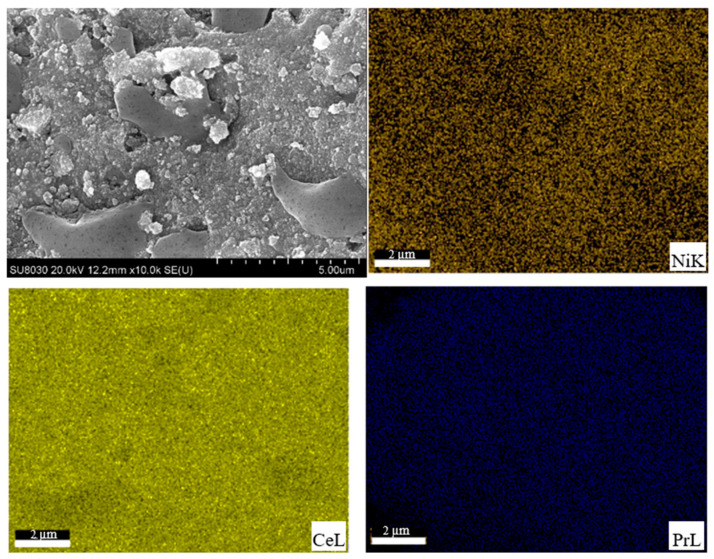
SEM image and elemental mapping of 5%Ni/Ce_0.95_Pr_0.05_O_1.975_.

The chemical analysis by SEM micrographs with the corresponding elemental mapping was conducted to investigate the elemental distribution and the homogeneity of the supported Ni catalyst. As presented in Figure 2, Ce, Pr, and Ni elements were uniformly distributed on 5%Ni/Ce_0.95_Pr_0.05_O_1.975_ catalysts. The highly dispersed Ni suggests a strong metal–support interaction, and the enhancement in Ni dispersion would provide more active sites that are exposed to reactants, which is beneficial to the increase in WGS performance.

Raman spectroscopy was performed to quantify oxygen vacancies in the catalyst. CeO_2_ catalyst initiates the water–gas shift process via a redox mechanism at high temperatures. CO adsorbs on the catalyst surface and subsequently oxidizes it with CeO_2_ lattice oxygen to generate carbon dioxide and oxygen vacancy. H_2_O oxidizes reduced CeO_2_ again to produce H_2_. A mechanism for increasing the catalytic performance of CeO_2_ is the incorporation of dopant ions, with Pr^3+^ as a promising candidate dopant [41]. It is widely regarded that when doping CeO_2_ with a trivalent cation, two Ce^4+^ ions in the CeO_2_ lattice are substituted by the dopants, and then an O ion is eliminated to conserve the charge [15]. Therefore, oxygen vacancy is directly related to catalytic activity in water–gas shift reaction.

**Figure 3:**
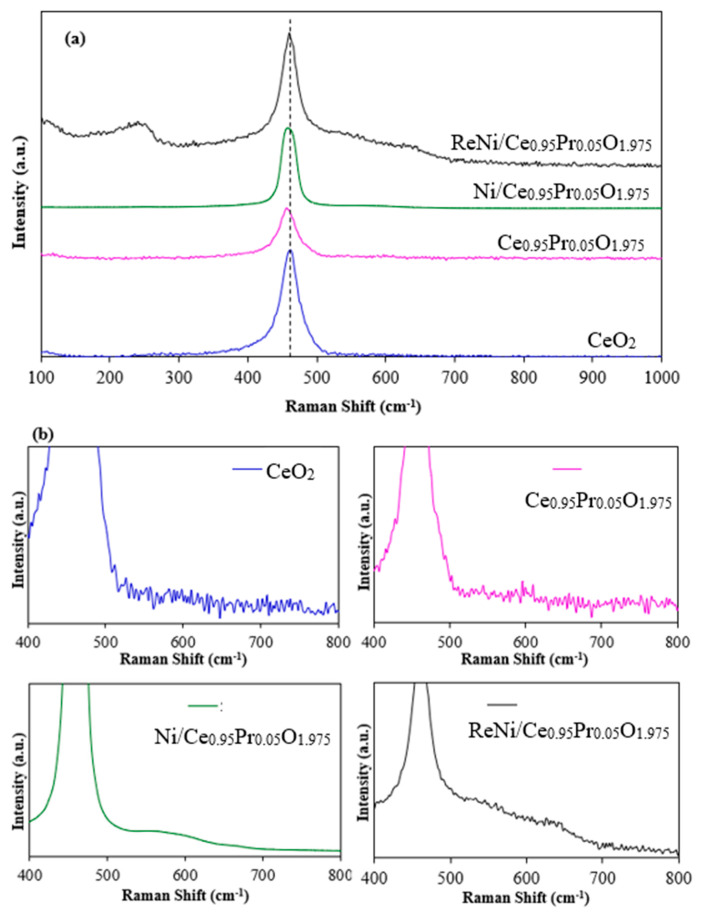
Raman spectra of CeO_2_ support and Ni catalysts in the wide range of 100–1000 cm^−1^ (**a**) and the narrow range of 400–800 cm^−1^ (**b**).

As shown in Figure 3, a Raman peak near 460 cm^−1^ was attributed to a triple degeneracy active mode (F_2g_ peak), which represents the symmetrical stretching vibration generated by eight O atoms bound to one Ce atom. Secondary peaks at around 240 and 320 cm^−1^ characteristics of CeO_2_ nanostructures are also found in 1%Re4%Ni/Ce_0.95_Pr_0.05_O_1.975_. In addition, another broad peak near 570 cm^−1^ (denoted by D peak) was associated with oxygen vacancies in CeO_2_ [42,43]. The oxygen vacancies concentration can be represented by the ratio of I_D_/I_F2g_ [44]. The intensity of the D peak in 1%Re4%Ni/Ce_0.95_Pr_0.05_O_1.975_ catalyst is stronger than that of other catalysts, indicating that higher oxygen vacancy concentration can be obtained by the addition of Re onto Ni/Ce_0.95_Pr_0.05_O_1.975_ catalyst. Moreover, the presence of Ce^3+^ in the CeO_2_-based catalyst can be demonstrated by a red shift of the F_2g_ peak, which is due to the lattice expansion when Ce^4+^ ions (ionic radius 0.097 nm) are replaced by Ce^3+^ ions (ionic radius 0.114 nm) for oxygen vacancy formation [43,45]. The enhancement of oxygen vacancy concentration in the 1%Re4%Ni/Ce_0.95_Pr_0.05_O_1.975_ catalyst enables the interaction between rhenium, nickel, and CeO_2_ to drive the metal dispersion and prevent the sintering of metal particles. This result indicates that the addition of Re to the Ni/Ce_0.95_Pr_0.05_O_1.975_ catalyst improves the reducibility and stability.

**Figure 4:**
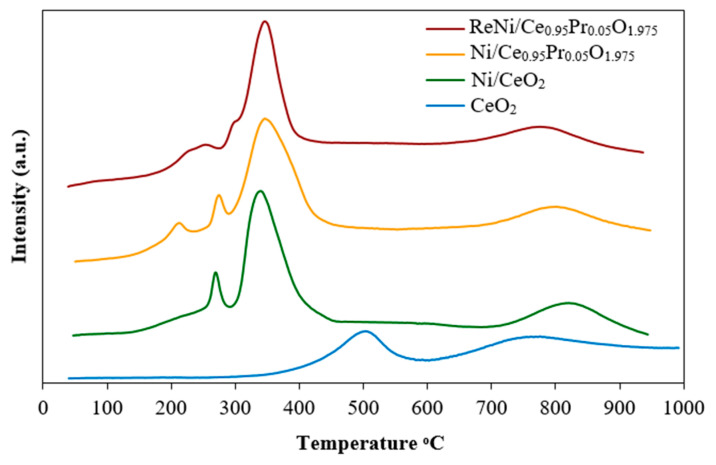
H_2_-TPR profiles of monometallic Ni and bimetallic NiRe supported by pure CeO_2_ and Pr-doped CeO_2_.

The H_2_-TPR profiles of CeO_2_, 5%Ni/CeO_2_, 5%Ni/Ce_0.95_Pr_0.05_O_1.975_, and 1%Re4%Ni/Ce_0.95_Pr_0.05_O_1.975_ catalysts are shown in Figure 4. The H_2_-TPR of CeO_2_ exhibits two broad peaks at 500 °C and 750 °C. The peak at 500 °C is assigned to the reduction of surface-capping oxygen of CeO_2_, and the peak at 750 °C is assigned to the bulk CeO_2_ reduction. The TPR profile of the Ni/CeO_2_ catalyst is characterized by a low-temperature peak at 272 °C, medium temperature at 345 °C, and bulk reduction at 830 °C. The reduction peak at 272 °C is assigned to the reduction of nickel oxide species. The consumption peak at 345 °C is assigned to the Ni-catalyzed reduction of the CeO_2_ surface [46,47]. It is interesting to note that the incorporation of Ni to CeO_2_ support significantly shifts the reduction peak of surface CeO_2_ from 500 °C to 345 °C. The H_2_-TPR of Ni/Ce_0.95_Pr_0.05_O_2−δ_ presented two nickel oxide reduction peaks at 220 °C and 278 °C, which was due to the different environments of Ni. The peak at 220 °C is probably due to the reduction of Ni in the vicinity of CeO_2_, whereas the consumption peak at 278 °C is due to the presence of Pr. The reduction peak of surface and bulk species of Ni/Ce_0.95_Pr_0.05_O_1.975_ appeared at the same position as the reduction of Ni/CeO_2_. This indicated that the addition of Pr to Ni/CeO_2_ alters the NiO reduction behavior. The H_2_-TPR profile of the bimetallic ReNi is different from those of monometallic Ni supported on Pr-doped CeO_2_. In this case, electron density transfers between Re, Ni, Ce, and Pr may occur. As the result of electron density transfer, a concurrent reduction of metal oxide species was found, and reduction of metal oxide is easier. A stronger interaction between nickel and CeO_2_ is expected to tune the nickel dispersion. The reduction peak appearing at a higher temperature normally means that it is more difficult to reduce with stronger metal–support interactions [48]. For the 1%Re4%Ni/Ce_0.95_Pr_0.05_O_1.975_ catalyst, stronger metal-support interactions are presented, which proved that the reduction of the surface shell of CeO_2_ occurred at a higher temperature. The stronger interaction between metal and support is beneficial to maintain the metal dispersion and hinder its aggregation. 

**Figure 5:**
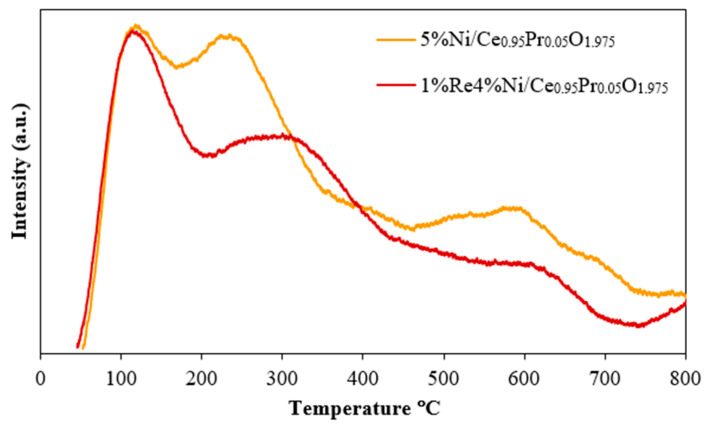
NH_3_-TPD of monometallic Ni and bimetallic NiRe supported by Pr-doped ceria.

The surface acidity of the prepared catalysts was studied using temperature-programmed desorption of ammonia (Figure 5), and the total acidity was estimated from the area under the NH_3_ desorption peak. NH_3_-TPD analysis was carried out in order to clarify the effect of the acidity of ReNi/Ce_0.95_Pr_0.05_O_1.975_ and Ni/Ce_0.95_Pr_0.05_O_1.975_ on the catalytic performance in the water–gas shift reaction. The peaks were assigned to weak, medium, or strong acid sites when falling in the 100–200 °C, 200–450 °C, or 450–700 °C temperature range, respectively. Increased surface acidity enabled a higher content of CO adsorption on the catalyst surface since a CO reactant in the WGS process is a weak base, explaining the observed increase in catalyst activity. Furthermore, the acidic character of the Ni catalyst surface proved to be beneficial for CO_2_ desorption, leaving behind free active sites for carbon monoxide and H_2_O adsorption in subsequent reaction cycles [49]. The result from NH_3_-TPD analysis indicates that the addition of Re onto Ni/Ce_0.95_Pr_0.05_O_1.975_ increases the concentration of weak-strength acid sites (peak area increases at <200 °C). In addition, the total concentration of surface acid sites can be estimated by integrating the NH_3_-TPD curves, and it was found to be 41 and 28 mols/g for 1%Re4%Ni/Ce_0.95_Pr_0.05_O_1.975_ and 5%Ni/Ce_0.95_Pr_0.05_O_1.975_, respectively. The obtained results could imply a higher tendency for carbon monoxide adsorption and subsequently easier CO_2_ desorption on the bimetallic ReNi supported by Pr-doped CeO_2_ surface, thereby the overall water–gas shift reaction rate over ReNi/Ce_0.95_Pr_0.05_O_1.975_ may be enhanced.

**Figure 6:**
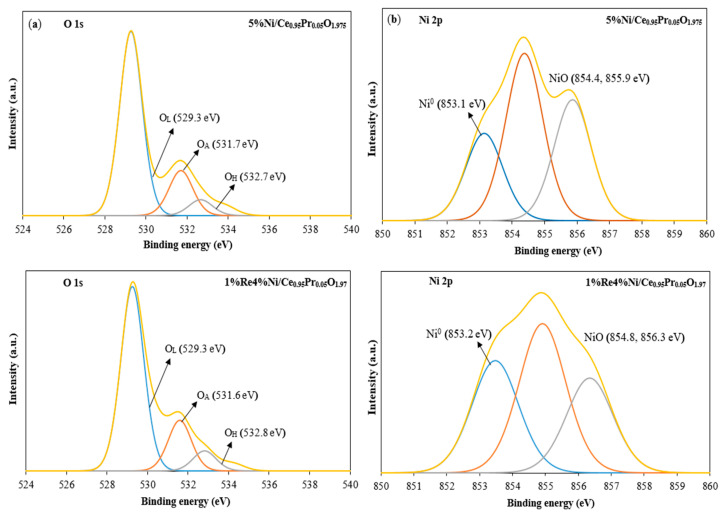
XPS spectra of reduced catalysts before the reaction in 5%Ni/Ce_0.95_Pr_0.05_O_1.975_ and 1%Re4%Ni/Ce_0.95_Pr_0.05_O_1.975_ for O 1s (**a**) and Ni 2p (**b**).

XPS characterization was used to investigate the surface chemical states of the catalysts. Figure 6a shows the O 1s XPS spectra of 5%Ni/Ce_0.95_Pr_0.05_O_1.975_ and 1%Re4%Ni/Ce_0.95_Pr_0.05_O_1.975_ catalysts. Three different types of oxygen species were detected in all samples. The detected peaks near 529 eV (O_L_), 532 eV (O_A_), and 533 eV (O_H_) are attributed to lattice oxygen in metal oxide, chemically adsorbed oxygen on the surface, and a surface hydroxyl oxygen species, respectively. The ratios of O_A_/O_L_, which are calculated from the area of each peak, are an indicator of active oxygen vacancies on the surface [50,51]. XPS results indicated that active oxygen vacancies were higher for the 1%Re4%Ni/Ce_0.95_Pr_0.05_O_1.975_ (O_A_/O_L_ = 0.28) compared to 5%Ni/Ce_0.95_Pr_0.05_O_1.975_ catalysts (O_A_/O_L_ = 0.24). Therefore, the bimetallic ReNi catalyst tended to display greater activity due to it producing more vacancies or defects. 

5%Ni/Ce_0.95_Pr_0.05_O_1.975_ and 1%Re4%Ni/Ce_0.95_Pr_0.05_O_1.975_ illustrated Ni 2p spectra mainly contributed by Ni^2+^ species at around 855 and 856 eV with a minor content of Ni^0^ species at around 853 eV (Figure 6b). All samples were reduced with 5%H_2_/N_2_ at 400 °C for 1 hour before XPS measurement. Ni species in the reduced catalysts were in the form of metallic Ni. Furthermore, the different Ni species co-existed due to the interaction with _CeO2_-based materials. Metallic Ni^0^ was indicated to be the dominant active species in accelerating the reactants with content of 36.6% and 23.3% in 1%Re4%Ni/Ce_0.95_Pr_0.05_O_1.975_ and 5%Ni/Ce_0.95_Pr_0.05_O_1.975_ catalysts, respectively. Therefore, the increase in metallic Ni amount in the bimetallic ReNi catalyst implies a superior catalytic performance of Ni catalyst by the addition of Re.

**Figure 7:**
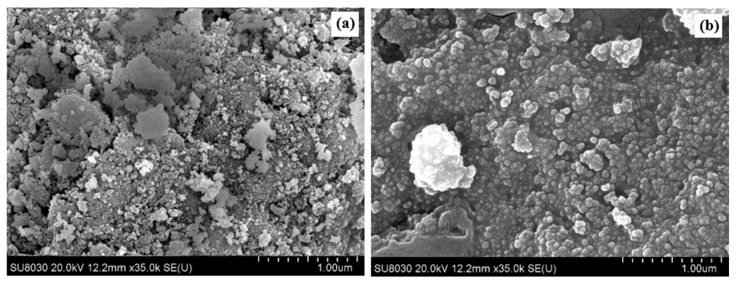
SEM results of (**a**) fresh and (**b**) used 1%Re4%Ni/Ce_0.95_Pr_0.05_O_1.975_ catalysts.

SEM analysis (shown in Figure 7) confirms there was almost no carbon deposition on the surface for the used 1%Re4%Ni/Ce_0.95_Pr_0.05_O_1.975_ catalyst. Wang et al. [52] reported that more carbon was deposited and accumulated on the surface of monometallic Ni catalysts during steam reforming of biomass tar, whereas bimetallic NiFe catalysts suppressed the carbon deposition on the surface of the reacted catalyst. Therefore, using bimetallic catalysts could prevent coke formation on the catalysts by providing oxidation of the accumulated carbon.

**Figure 8:**
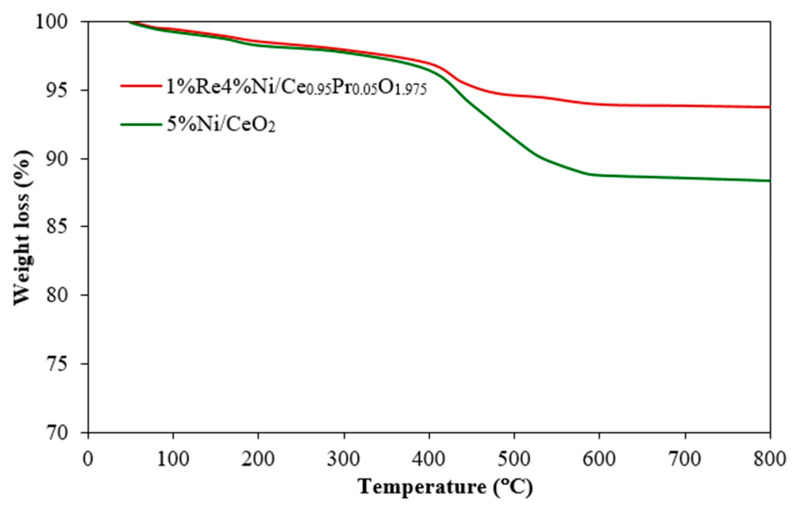
Thermo-gravimetric analysis of the used catalysts.

The carbon deposition of the spent catalysts was evaluated by TG analysis (Figure 8). The oxidation of the carbon deposition in the air leads to weight loss. Small weight loss at low temperatures (below 200 °C) was ascribed to the elimination of moisture and volatile species [53]. The mass loss in the range of 200–400 °C was ascribed to the thermal decomposition of physisorbed carbonaceous species or soft-coke. A major weight reduction between 400 and 600 °C was due to the bulky carbonaceous products or hard coke on the used catalysts. The weight loss percentages of the bulky carbonaceous species on monometallic Ni and bimetallic NiRe catalysts were 11.6% and 6.2% for 5%Ni/CeO_2_ and 1%Re4%Ni/Ce_0.95_Pr_0.05_O_1.975_, respectively, indicating that carbon decomposition decreases when use Pr as dopant and Re as metal additives. 

### 2.2. Water–Gas Shift Activity and Stability

**Figure 9:**
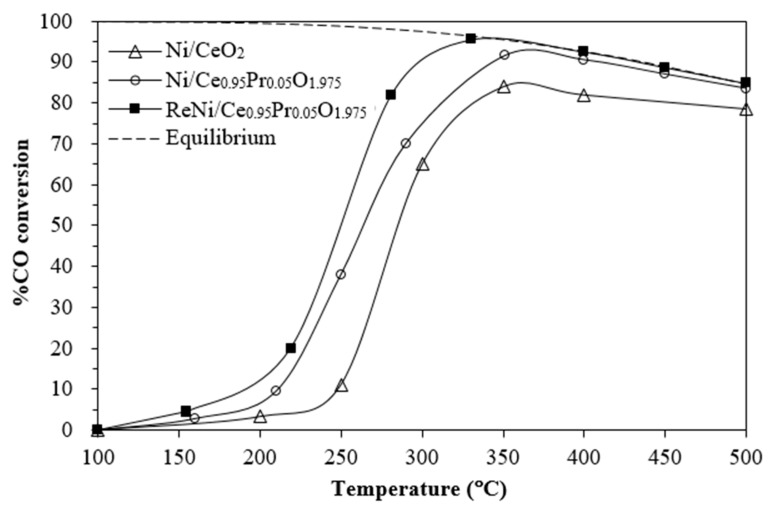
%CO conversion of supported Ni catalysts.

Figure 9 exhibits the %CO conversion of Ni/CeO_2_, Ni/Ce_0.95_Pr_0.05_O_1.975_, and ReNi/Ce_0.95_Pr_0.05_O_1.975_. From previous studies, it was found that further addition of Re does not further raise the rate of water–gas shift reaction, and the optimal content of Re that is enough to maximize the water–gas shift rate is 1 %wt. [54]. When attention is drawn to the variation in Sm amount [12], it appears that the Ni catalyst with 5%Sm-doped CeO_2_ gives the highest water–gas shift activity. The enhancement of Sm content to 15 wt.% leads to a lowering of nickel dispersion. This result is due to the agglomeration of samarium at a high amount. Therefore, 5% doping amount and 1% of Re metal additives were used in this work to maximize the WGS performance.

For Ni/CeO_2_, the CO conversion started above 150 °C and ascended slowly to reach the maximum conversion of 84% at 350 °C. As observed, the highest CO conversion was achieved over a bimetallic NiRe/CePrO catalyst, reaching 96% CO conversion at 330 °C with a WGS activity higher than the activities of the monometallic catalysts. NiRe/Ce_0.95_Pr_0.05_O_1.975_ has been determined as an excellent catalyst due to its high surface acidity, nickel metal dispersion, and nickel surface area which can enhance the concentration of CO adsorption on the catalyst surface. Furthermore, the NiRe/Ce_0.95_Pr_0.05_O_1.975_ catalyst produced more oxygen vacancies, which could increase the redox ability, causing higher WGS activity. 

**Figure 10:**
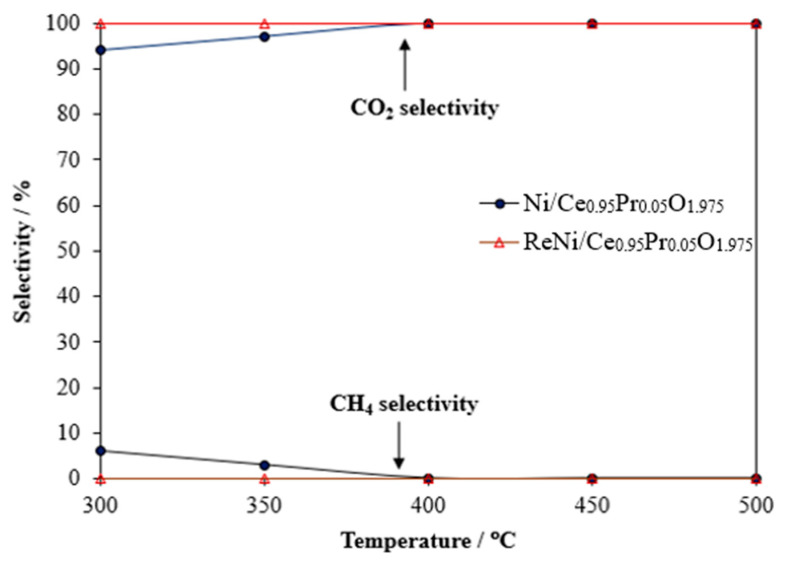
Selectivity to CO_2_ and CH_4_ as a function of temperature over supported Ni catalysts.

Figure 10 presents the CO_2_ and CH_4_ selectivity of Ni/Ce_0.95_Pr_0.05_O_1.975_ and ReNi/Ce_0.95_Pr_0.05_O_1.975_ in the temperature range of 300–500 °C. 1%Re4%Ni/Ce_0.95_Pr_0.05_O_1.975_ was exhibited to be an excellent catalyst in terms of WGS activity and selectivity of CO_2_ and CH_4_. Methane is an unwanted product because it is a precursor for coke formation and competes against H_2_ generation. As observed, 5%Ni/Ce_0.95_Pr_0.05_O_1.975_ generates CH_4_ at low temperatures, whereas 1%Re4%Ni/Ce_0.95_Pr_0.05_O_1.975_ is highly selective toward the WGS reaction throughout the investigated temperature. Thus, the incorporation of Re onto Ni/Ce_0.95_Pr_0.05_O_1.975_ increased CO conversion at the same time that it suppressed CH_4_ formation.

**Figure 11:**
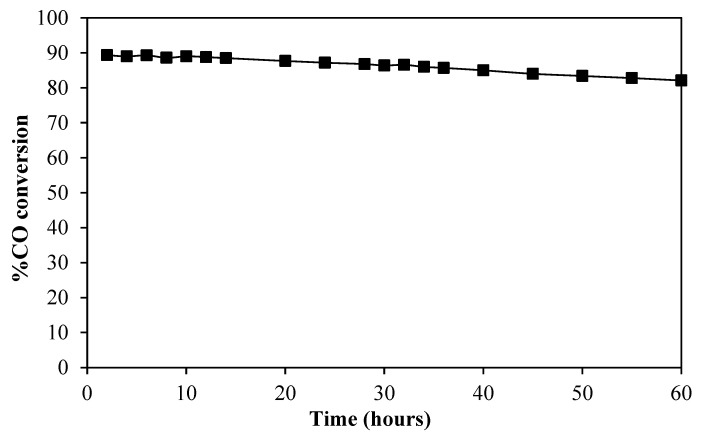
Long-term stability test at 300 °C of 1%Re4%Ni/Ce_0.95_Pr_0.05_O_1.975_.

The water–gas shift stability has been performed on the most active catalyst, ReNi/Ce_0.95_Pr_0.05_O_1.975_, under the feed mixture containing 5% CO, 10% H_2_O, and 85% N_2_ at 300 °C. As shown in Figure 11, the ReNi/Ce_0.95_Pr_0.05_O_1.975_ catalyst retained a high CO conversion of about 89% during the first 20 h on stream. Then, the CO conversion slightly decreases to 82% after 60 h of reaction. Hence, the bimetallic ReNi supported by Pr-doped CeO_2_ is resistant toward deactivation during a water–gas shift reaction.

## 3. Experimental Procedure 

### 3.1. Catalysts Preparation

A combustion technique was used to synthesize pure CeO_2,_ and *ceria*–praseodymia mixed oxides supports with Ce(NO_3_)_3_.6H_2_O (Sigma-Aldrich, Pte. Ltd., Singapore) and Pr(NO_3_)_3_.6H_2_O (Sigma-Aldrich, Pte. Ltd., Singapore) as starting materials. Urea was utilized as a fuel to ignite the reaction. The redox reactions between NH_2_CONH_2_ and metal nitrates provide the exothermicity essential for the nucleation and growth of the metal oxide powders [55]. Metal nitrate was mixed with urea using a stoichiometry between urea and metal nitrates as 1:2.5. Stirring a mixture obtained a homogeneous solution and then heating with a Bunsen burner until autoignition occurred. CeO_2_ and ceria–praseodymia mixed oxides powders were obtained by the thermal decomposition of nitrate and other organic compounds [56]. 

Ni(NO₃)₂.6H₂O (Alfa Aesar, Thermo Fisher Scientific Inc, Seoul, Republic of Korea) and NH_4_ReO_4_ (Sigma-Aldrich, Pte. Ltd., Singapore) were used as the metal precursors for the preparation of Ni/CeO_2_, Ni/Ce_0.95_Pr_0.05_O_1.975_, and ReNi/Ce_0.95_Pr_0.05_O_1.975_ via impregnation method. A solution of nickel and rhenium was added to ceria and ceria–praseodymia mixed oxides powders. All catalysts were dried at 100 °C for 12 h and calcined at 650 °C for 8 h.

### 3.2. Catalyst Characterization

The specific surface areas of all catalysts were measured by N_2_ adsorption–desorption isotherms at 77 K using the BELSORP-MAX instrument (ITS Co. Ltd., Bangkok, Thailand). The samples were outgassed at 300 °C for 3 hours before the analysis. The Brunauer–Emmett –Teller method was utilized to estimate the specific surface areas of the catalysts.

X-ray powder diffraction (XRD) was performed using a PANalytical X’Pert Pro diffractometer (Malvern Panalytical Ltd., Malvern, UK) with the filtered radiation of a copper anode in the range temperature of 20–80°. The X-ray diffractograms were collected using the current of 40 mA and 40 kV with 0.02° per step and 0.5 s per step. The crystallite sizes of CeO_2_ were estimated from the full width at half maximum of the strongest (111) reflection using the Debye–Sherrer equation.

Raman spectra were collected on Perkin-Elmer System 2000 FTIR/FT-Raman (Perkin Elmer, Rodgau, Germany) with argon ion laser irradiation at 532 nm wavelength and 10 mW maximum power. The spectra were recorded over the range of 100–1000 cm^−1^ using an operating spectra resolution of 1.0 cm^−1^ of Raman shift. 

The H_2_ chemisorption, Temperature Programmed Desorption of Ammonia (NH_3_-TPD), and H_2_-Temperature Programmed Reduction (H_2_-TPR) were performed using a catalyst analyzer BELCAT-B instrument (ITS Co. Ltd., Bangkok, Thailand) equipped with a thermal conductivity detector. The reduction behavior of the samples was studied by H_2_-TPR. The catalyst was first heated from room temperature to 120 °C in the He flow, maintained at 120 °C for 30 min, and cooled down to 50 °C under the He flow. The TPR measurement was performed from 50 °C up to 1000 °C with the rate of 10 °C/min under 5%H_2_ in argon flow. NH_3_-TPD analysis was performed to investigate catalyst acidity. The catalyst was first heated from room temperature to 500 °C in argon flow and cooled down to 50 °C under argon. The catalyst was then exposed to pulse titration by using a loop of a known volume of NH_3_ in Ar flow until saturation. NH_3_-TPD was finally carried out from 50 to 800 °C with a heating rate of 10 °C/min under argon flow. The H_2_ chemisorption was performed to determine the surface area, particle size, and dispersion of Ni metal. The sample was evacuated in the He flow at 40 °C and then reduced at 400 °C for 1 h under H_2_ flow (30 mL/min). The reduced catalyst was cooled down to 40 °C under helium flow and followed by volumetric H_2_ chemisorption with pure hydrogen. The Ni surface area (S_Ni_), Ni dispersion (D), and Ni particle size (d_Ni_) were obtained from the instrument software based on the calculation by the following equation [57,58].

D (%) = V_H_ × M_Ni_ × F/Vm × W × 100 (2)

S_Ni_ (m^2^/g) = V_m_ × N_A_ × F × A_Ni_/V_molar_ (3)

d_Ni_ = 60 × W/ρ × S_Ni_ (4)

where V_H_ is the chemisorbed H_2_ volume (mL/g), Vm is the molar volume of H_2_ (mL/mol), W is % wt. of nickel, M_Ni_ is the atomic weight of nickel (g/mol), A_Ni_ is the cross-sectional area of nickel atom (m^2^/atom), ρ is the density of Ni (g/mL), N_A_ is Avogadro’s number, and F is the stoichiometry factor (the number of active metal atoms to which one adsorbate gas molecule can attach).

Scanning electron microscopy (SEM) was performed on an FE-SEM (HITACHI SU-8030, Hitachi High-Technologies Corporation, Tokyo, Japan) with high vacuum mode using secondary electrons and an acceleration of 30 kV. Energy dispersive X-ray spectroscopy (EDX) was used in conjunction with scanning electron microscopy for the elemental analysis. 

Thermo-gravimetric analysis was performed using a Perkin-Elmer TGA/DTA 6300 instrument (Perkin Elmer, Rodgau, Germany) under an airflow rate of 100 mL/min. The content of carbon deposition on the used catalysts was investigated. The mass change in Ni-based catalysts was measured as a function of temperatures up to 800 °C with a heating rate of 20 °C/min. 

### 3.3. Water–Gas Shift Activity

The water–gas shift activity was measured at the temperature from 100 to 500 °C. The catalyst (150 mg) was placed inside a fixed bed flow reactor (310 stainless steel, 0.6 cm outside diameter) between two layers of quartz wool. The catalyst was reduced under 5% H_2_ in N_2_ flow at 300 °C for an hour before the WGS activity testing. H_2_O was fed through a pre-heater using a syringe pump, whereas the flow rates of CO and N_2_ were controlled by a mass flow controller. A mixed gas containing 5% CO, 10% H_2_O, and 85% N_2_ was fed into the reactor. The total flow rate was maintained at 100 mL/min in all testing conditions. The composition of the gas mixture leaving the reactor was determined using an online Shimadzu GC-14B gas chromatography equipped with a thermal conductivity detector (TCD) and a ShinCarbon ST column. Argon is used as the eluent for a ShinCarbon ST column to detect the H_2_, CO, and CH_4_ at the rate of 50 mL/min. The concentration of CO, CO_2_, and CH_4_ at the outlet was repeated at least four times for each analysis. The water–gas shift activities can be calculated according to the following equation:

(5) $$\%\mathrm{CO}\;conversion=\frac{CO_{in}-CO_{out}}{CO_{in}}\times 100$$

where CO_in_ is the inlet molar flow rate of CO (mol s^−1^) and CO_out_ is the outlet molar flow rate of CO (mol s^−1^).

## 4. Conclusions

The influence of Re and Pr on the catalytic activity of Ni/CeO_2_ was studied. The incorporation of Re and Pr into Ni/CeO_2_ increased the WGS efficiency when compared with Ni/CeO_2_. An addition of Pr to Ni/CeO_2_ reduced the crystallite size of CeO_2_, increased the BET surface area, and promoted higher dispersion of nickel on the CeO_2_ surface. Furthermore, the role of rhenium on the water–gas shift performance of supported Ni catalyst was also considered. The results revealed that the addition of rhenium onto Ni/Ce_0.95_Pr_0.05_O_1.975_ increased the catalytic performance toward the water–gas shift reaction and suppressed CH_4_ formation. The role of rhenium in improving the catalytic activity was due to an increase in surface acidity, Ni surface area, and Ni dispersion, which facilitate CO adsorption on the catalyst surface. Additionally, the acidic character of the catalyst can accelerate CO_2_ desorption, leaving behind free active sites for the adsorption of CO and H_2_O reactants in subsequent reaction cycles. Moreover, the enhancement of oxygen vacancy concentrations alerts the redox processes at the catalyst surface, which contributes to improving the WGS rate.

## Figures and Tables

**Table 1:** BET surface area, crystallite size of CeO_2_, Ni dispersion, and Ni surface area of Ni-based catalysts.

Catalysts	Crystallite Size ^a^ (nm)	BET Surface Area ^b^ (m^2^/g)	Ni Dispersion ^c^ (%)	Ni Particle Size ^c^ (nm)	Ni Surface Area ^c^ (m^2^/g)
CeO_2_	9.8	68	-	-	-
5%Ni/CeO_2_	13.35	45	0.17	35.1	0.95
5%Ni/CePrO	8.55	64	0.30	19.6	1.70
1%Re4%Ni/CePrO	8.01	60	1.15	5.50	6.08

^a^ Calculated from the 111 diffraction peak broadening. ^b^ Estimated from N_2_ adsorption at −196 °C. ^c^ Estimated from H_2_-chemisorption.

## Data Availability

Data are contained within the article.
